# Effects of *L. plantarum* HY7715 on the Gut Microbial Community and Riboflavin Production in a Three-Stage Semi-Continuous Simulated Gut System

**DOI:** 10.3390/microorganisms9122478

**Published:** 2021-11-30

**Authors:** Dong-Ki Hong, Myeong-Seok Yoo, Keon Heo, Jae-Jung Shim, Jung-Lyoul Lee

**Affiliations:** R&BD Center, hy Co., Ltd., 22, Giheungdanji-ro 24beon-gil, Giheung-gu, Yongin-si 17086, Korea; dkhong@hy.co.kr (D.-K.H.); audtmrl@hy.co.kr (M.-S.Y.); jjshim@hy.co.kr (J.-J.S.); jlleesk@hy.co.kr (J.-L.L.)

**Keywords:** *L. plantarum* HY7715, riboflavin, gut microbiome, butyrate, simulated gut system

## Abstract

Probiotics should be well established in the gut, passing through the digestive tract with a high degree of viability, and produce metabolites that improve the gut environment by interacting with the gut microbiome. Our previous study revealed that the *Lactiplantibacillus plantarum* HY7715 strain shows good bile acid resistance and a riboflavin production capacity. To confirm the interaction between HY7715 and gut microbiome, we performed a metabolite and microbiome study using a simulated gut system (SGS) that mimics the intestinal environment. Changes in the microbiome were confirmed and compared with *L. plantarum* NCDO1752 as the control. After 14 days, the HY7715 treatment group showed a relatively high butyrate content compared to the control group, which showed increased acetate and propionate concentrations. Moreover, the riboflavin content was higher in the HY7715 treatment group, whereas the NCDO1752 treatment group produced only small amounts of riboflavin during the treatment period and showed a tendency to decrease during the washout stage; however, the HY7715 group produced riboflavin continuously in the ascending colon during the washout period. A correlation analysis of the genus that increased as the content of riboflavin increased revealed butyrate-producing microorganisms, such as *Blautia* and *Flavonifractor*. In conclusion, treatment with *L. plantarum* HY7715 induced the production and maintenance of riboflavin and the enrichment of the intestinal microbiome

## 1. Introduction

Probiotics are non-pathogenic microorganisms that have a beneficial effect on the gut health of hosts such as humans and animals, most probiotics being lactic acid bacteria, and some including Bacillus and yeast, and the World Health Organization (WHO) defines a probiotic as “a microorganism conferring a health benefit to the host when administered in appropriate amounts” [1]. Various studies have reported the functionalities of probiotics, which include the maintenance of basic intestinal and live health and the prevention of conditions such as bloating, lower abdominal pain, diarrhea and non-alcoholic hepatitis through interactions with the intestinal microbiome after ingestion, and cognition and brain health associated with Alzheimer’s disease [2,3,4,5,6,7]. The scope of functional research and product applications is expected to continue to expand in the future. In general, lactic acid bacteria are Generally Recognized as Safe (GRAS), so it is easy to commercialize them as probiotics if they prove beneficial effects as probiotics. These bacteria are typically consumed in fermented milk products and probiotic powders [8]. However, in order for lactic acid bacteria to be accepted as probiotics and produce advantageous affects, they must survive stomach acid and bile acid activity, reach the small intestine, and establish and multiply [9,10] In addition, the European Food Safety Authority requires that probiotics be validated in human studies that are reproducible, documented, and studied not only for the basic characteristics of probiotics, but also for their overall consumer health benefits [11].

However, experiments on human subjects are expensive and time consuming, and it is virtually impossible to control individual diets and lifestyles. In particular, interpreting changes in the microbiota of feces is highly complex. For example, the bacterial composition of the initial colony of a newborn’s feces varies depending on whether the child was delivered naturally or via cesarean section [12]. Moreover, differences in the bacterial composition of adult feces are influenced by protein versus vegetarian diets, climate and weather, and the medical history [13,14,15]. Although microbiome studies and probiotics efficacy studies can be conducted using humanized microbiota mice, only a portion of human microbiota is successfully transferred to mice and, thus, it is not an accurate representation of the human microbiome [16,17]. Microbiome research using a simulated gut model is one method to overcome these limitations. The human gut microbiota comprises 10^10^–10^12^ cells/g of feces and approximately 400 species of bacteria from the ecosystem in a symbiotic relationship with the host [18,19,20]. Gut microbes play important roles in the enzymatic digestion of food residues and are major contributors to human health. In vitro gut fermentation models can be used as representatives of the human intestinal ecosystem, which involve inoculating human feces with defined volumes of media under controlled temperature and pH conditions in either a static or continuous culture system [18,21]. A batch-type model is a closed system in which nutrients are not replenished and waste is not removed after the initial inoculation, making it suitable for the short-term analysis of simple gut chemicals such as oligosaccharides and phytochemicals [22,23]. Conversely, a continuous model involves replenishing the system with fresh media and continuously discharging waste, allowing for the long-term study of more complex chemicals [24]. In addition, the use of a continuous system allows for observation of the compositional changes in metabolites and microbial communities [25] as well as the analysis of the three subdivisions of the large intestine, namely, the ascending colon (AC), transverse colon (TC), and descending colon (DC).

Riboflavin is an essential vitamin that plays an important role in the human diet and health. Riboflavin is known to play an important role as an essential element in mitochondrial energy metabolism, stress response, and metabolic cofactors [26]. Moreover, the anti-inflammatory effects of riboflavin via the lowering of several anti-inflammatory cytokines, namely, TNF-α, IL-1ß, and IL-6, have also been reported [27,28]. Although riboflavin is abundant in dairy products and meat, deficiency can occur even with a sufficient intake. Deficiency factors are associated with birth genetic disorders, infection, exercise, diet, aging, and pregnancy, or with environmental and physiological factors [26]. Therefore, because of the significant impacts of this compound on human gut health, it is necessary to study the relationship between riboflavin and the intestinal microbiome.

Studies on the selection and effectiveness of probiotic strains with an excellent riboflavin-producing ability have been reported, and the production of riboflavin after intestinal settlement through probiotics has been reported to have a double effect on the basic functionality of probiotics [29,30]. In our previous studies, we demonstrated the vitamin B2 production ability and high survival rate of the *L. plantarum* HY7715 (HY7715) strain against gastric and bile acids, and the safety of the HY7715 strain was confirmed through Minimum Inhibitory Concentration (MIC) and hemolysis tests [31].

In this study, we analyze changes in intestinal microflora induced by the HY7715 strain in a simulated gut model. Moreover, we compare changes in the fatty acid (basic metabolite) and riboflavin contents through intestinal colonization relative to the type of strain, and analyze the influence of probiotics on the gut microbiome. Finally, we confirm the possibility of grafting in probiotic functional studies using the simulated gut model of the human gut microbiome in the screening stage before conducting animal experiments.

## 2. Materials and Methods

### 2.1. Gut Culture System Simulation

**Figure 1:**
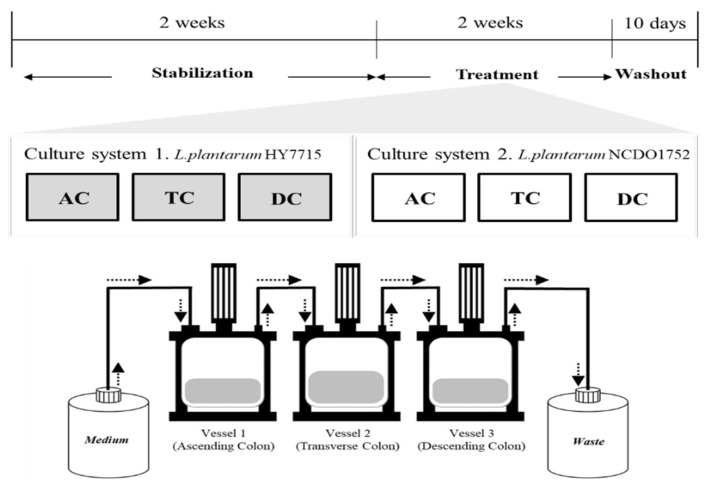
Schematic diagram of the continuous culture system employed in this study: AC—ascending colon; TC—transverse colon; DC—descending colon.

Fecal samples were obtained from five volunteers who had not taken antibiotics for three months. No personal information, including the health status, age, or gender, was collected. The stool samples were diluted with phosphate-buffered saline (PBS) in an anaerobic chamber for 30 min. The fecal slurry was then inoculated into each vessel of the chamber at a final concentration of 2% (*w/v*). To stimulate intestinal microbiota growth, the system was operated under static conditions of 37 °C, pH 5.7, and 200 rpm for the first 24 h. Continuous flow was sustained by adjusting the interval of the pump on/off time at 12.5 mL/h. The system was maintained under anaerobic conditions by the continuous injection of N_2_ gas (10 mL/min). The volume and pH condition of each vessel were adjusted to 300 mL, pH 5.5 for the Ascending Colon (AC); 400 mL, pH 6.2 for the Transversal Colon (TC), and 325 mL, pH 6.8 for the Descending Colon (DC). The culture medium contained 1 g/L peptone, 3 g/L yeast extract, 4 g/L mucin, 0.4 g/L d-glucose, 0.5 g/L l-cysteine-HCl, 2 g/L pectin, 1 g/L xylan, 1 g/L arabinogalactan, 0.5 g/L inulin, 3 g/L starch, 0.4 g/L bile salt, and 0.0025 g/L resazurin. The pH of each vessel was adjusted automatically by the addition of 1 *N* HCl and 1 *N* NaOH. Prior to the probiotic treatments, the continuous system was run in advance to achieve a microbial and chemical stationary phase until the SCFA content became convergent. A schematic diagram is shown in Figure 1.

### 2.2. Probiotics Treatment

*L. plantarum* HY7715 (HY7715) and *L. plantarum* DCDO 1752 (NCDO1752) were inoculated into an MRS broth medium (Difco Corp., Sparks, MD, USA) and cultured at 37 °C for 18–24 h. Each MRS culture medium was subcultured in the simulate gut system (SGS) medium and cultured for 18–24 h at 37°C; colony-forming units (CFUs) were measured. The cultured HY7715 and NCDO1752 in SGS medium were diluted to a concentration of 10^7^ CFU/mL per vessel and inoculated into the ascending colon section and treated daily for a 14 d period. After 14 d, the culture solution of the wash-out stage without lactic acid bacteria treatment was sampled for an additional 7 d. Each sample was immediately frozen in liquid nitrogen after collection and stored at −80 °C in a freezer until analysis.

### 2.3. Riboflavin Analysis

The riboflavin content analysis was performed using high-performance liquid chromatography (HPLC) combined with a fluorescence detector (Agilent-1100 Series, Palo Alto, CA, USA), as well as the C-18 column Unison UK-C18 (4 × 100 mm, 3 μm, Imtakt, Portland, OR, USA). The SGS culture medium was boiled at 100 °C for 15 min and then centrifuged at 13,000 rpm and 4 °C for 15 min. The supernatant was collected and filtered through a 0.45 μm membrane filter (Millipore, Burlington, MA, USA). Methanol (mobile phase A) and a 10 mM sodium dihydrogen phosphate (Na2HPO4) solution (pH 5.5, mobile phase B) were used as the mobile phases according to the following gradient conditions: 0 min—A 90%, B 10%; 10 min—A 60%, B 40%, 10.1 min A—90%, B 10%; 20 min—A 90%, B 10%. The column temperature, injection volume, flow rate, and excitation and emission wavelengths were 40 °C, 10 µL, 0.8 mL/min, 445 nm, and 530 nm, respectively. Supelco (Bellefonte, PA, USA), a riboflavin standard reagent, was used for riboflavin analysis, and the retention time and peak area was compared to quantify the riboflavin concentration of the sample.

### 2.4. Short-Chain Fatty Acid Measurements

The cultured samples were boiled for 10 min and centrifuged at 13,000 rpm for 15 min. The supernatant was then collected and filtered through a 0.45 μm filter. The SCFAs were determined using an HPLC system (Agilent Technologies) with an Aminex HPX 87-H column (Bio-Rad, Hercules, CA, USA) and a 215 nm UV detector. The column was eluted with 0.001 *N* H_2_SO_4_ at a flow rate of 0.6 mL/min.

### 2.5. Microbial Community Profiling

Metagenome analysis of SGS culture samples were carried out at Macrogen, Inc. (Seoul, Korea), and DNA was extracted using the DNeasy PowerSoil Pro Kit (Cat. no. 47016, QIAGEN) according to the manufacturer’s protocol. Each sequenced sample was prepared according to Illumina 16S Metagenomic Sequencing Library protocols. The quantification of DNA and DNA quality was conducted using PicoGreen and Nanodrop. PCR amplification was targeted to the V3 to V4 regions of the 16SrRNA with 341F (5’-TCGTCGGCAGCGTCAGATGTGTAT-AAGAGACAGCCTACGGGNGGCWGCAG-3’; the underlined sequence indicates the target region primer) and 805R (5’-GTCTCGTGGGCTCGGAGATGTGTATAAGAGACAGGCTACHVGGGTATCTAATCC-3’). The PCR amplifications were conducted under the following conditions: initial denaturation at 95 °C for 3 min followed by 25 cycles of denaturation at 95 °C for 30 s, primer annealing at 55 °C for 30 s, and extension at 72 °C for 30 s, with a final elongation at 72 °C for 5 min. Input gDNA was amplified with 16S V3–V4 primers, and a subsequent limited-cycle amplification step was performed to add multiplexing indices and Illumina sequencing adapters. The final products were normalized and pooled using PicoGreen, and the library sizes were verified using the LabChip GX HT DNA High Sensitivity Kit (PerkinElmer, Massachusetts, USA). Sequencing was performed using the MiSeq™ platform (Illumina, San Diego, USA). After trimming, a paired-end sequence was generated using the FLASH software (v. 1.2.11). Raw data were analyzed using the QIIME v. 1.9.0 program. Sequence data were filtered for poor quality reads, and unmatched indexes were truncated. Sequences were clustered into operational classification units (OTUs) with a 97% cutoff value using CD-HIT-OTU analysis. After OTU clustering, OTUs were sorted and assigned to the NCBI16S microbial database. Classification information was based on the BLAST + database (v. 2.9.0).

### 2.6. Statistical Analysis

The datasets were organized using Microsoft Office Excel (version 2014; Microsoft, Redmond, WA, USA) and the *R* program (version 4.1.1; https://cran.r-project.org/bin/windows/base, accessed on 9 July 2021). The data variances were analyzed using Student’s t-test for two groups and analysis of variance for multiple groups. Moreover, the K-paired Sample Friedman test was used to analyze the significance levels between samples of the large intestine and the treated bacterial species. The microbiome taxonomic profiling data were analyzed after normalization of the read count and gene copy number. Beta-diversity analysis was determined by principal coordinate analysis with the Jensen–Shannon divergence test, and the microbial flora composition was compared using the Wilcoxon rank-sum test. Differences were considered significant if the *p*-value was less than 0.05.

## 3. Results

### 3.1. SCFA Changes in the Culture System

**Figure 2:**
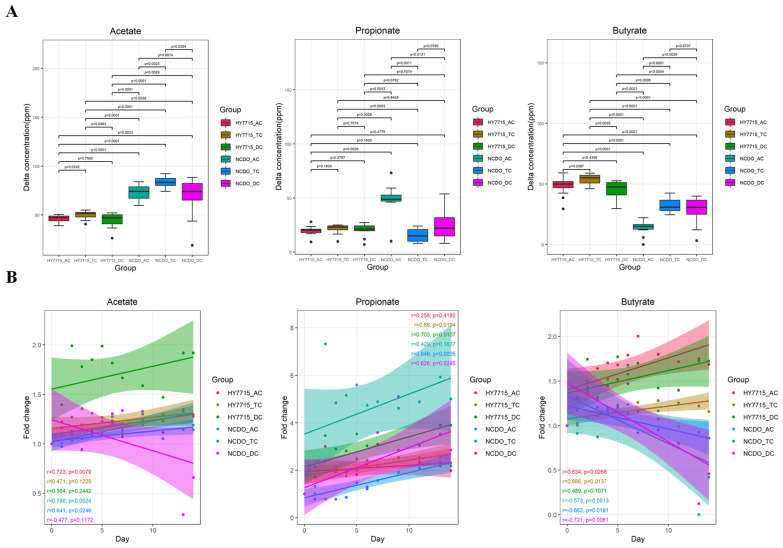
Changes in short-chain fatty acids (acetate, propionate, and butyrate) in the fecal inoculum with strains HY7715 and NCDO175: (**A**): boxplot of changes in the short-chain fatty acid content during the 14 d treatment period compared to the 0 day value of the HY7715 and NCDO treatment groups. Statistical analysis was performed using the Kruskal–Wallis nonparametric test. (**B**): Results of the correlation analysis of short-chain fatty acids vs. date fold change. The trend slope R-value indicates the correlation coefficient and the shaded range around the straight line is twice the standard deviation (AC—ascending colon; TC—transversal colon; DC—descending colon).

The fatty acid content, expressed as delta ppm, was determined by calculating the change in the content from immediately before treatment to after the treatment. Boxplots were used to analyze the change in the value of short-chain fatty acids during the probiotic treatment period from 0 to 14 d (Figure 2A). Furthermore, the Pearson correlation analysis conducted between the fold change of SCFAs and the treatment day to confirm an increase or decrease tendency of short-chain fatty acids in each AC, TC, and DC (Figure 2B). In the case of acetate, both the experimental and control group showed increasing tendencies, and it was confirmed that the HY7715 treatment group showed a greater increase in the DC than the AC. Propionate increased significantly in the NCDO1752 treatment group. In particular, the increase in propionate in the AC of the NCDO1752 treatment group was overwhelmingly high. The butyrate content increased in both the HY7715 and NCDO1752 treatments, and the increase in HY7715 was more significant in all three colon sections. In the case of the HY7715 treatment group, the average fold change values were 1.6, 1.2, and 1.5 in AC, TC, and DC after treatment, respectively. In the NCDO treatment group, the butyrate content increased in the initial stage of the treatment, but gradually decreased after 7 days of treatment, showing an average fold change value of 1.0~1.1 in the AC, TC, and DC. The lactate content was also analyzed, but no peak was detected in any of the samples. To analyze the correlation between the fatty acid content and the treatment date, the fatty acid content was divided by the initial value of each part and converted into a fold change for a relative comparison. For acetate, NCDO1752 and HY7715 showed relatively high positive correlations of 0.786 and 0.723, respectively, in the AC portion. The propionate content also showed a positive correlation with the treatment day in all regions of each treatment group, as well as a high positive correlation of 0.846, the highest in the NCDO1752 AC region. The fold change of butyrate was different from that of acetate and propionate. The HY7715 and NCDO1752 treatment groups showed a positive and negative correlation, respectively, with the treatment date (Figure 2B).

### 3.2. Riboflavin Production in the Culture System

**Figure 3:**
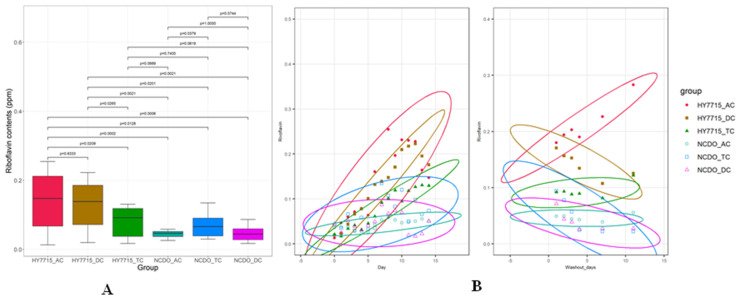
Graph of riboflavin concentration change according to HY7715 and NCDO1752 treatment days. (**A**): Boxplot of riboflavin concentration on treatment days (0–14 days) for each analyzed colon section. Statistical analysis was performed using the Kruskal–Wallis nonparametric test. (**B):** Increase/decrease trend graph with changes in the riboflavin concentration by treatment species, site, and date in the washout stage (right). Only HY7715 showed an increasing trend.

As a result of analyzing the riboflavin content for 14 d via daily treatment with lactic acid bacteria, we observed that the riboflavin content in the HY7715 treatment group was higher than that in the NCDO1752 treatment group (Figure 3A). In the HY7715 treatment group, the mean riboflavin content in the AC, TC, and DC regions was 0.14 ± 0.08 ppm, 0.13 ± 0.07 ppm, and 0.08 ± 0.04 ppm, respectively. Moreover, we observed the highest increase in riboflavin content in the HY7715 treatment AC portion, and the maximum value was 0.25 ppm. Conversely, in the NCDO1752 treatment group, the average riboflavin content in the AC, TC, and DC regions was 0.04 ± 0.01 ppm, 0.05 ± 0.02 ppm, and 0.07 ± 0.03 ppm, respectively, and the highest riboflavin concentration (0.13 ppm) was observed in the DC region. Comparing the daily riboflavin concentration change, we observed that the HY7715 treatment showed a sharper increase than the NCDO1752 treatment group. In addition, in the washout stage, the riboflavin content was maintained in the AC portion of the HY7715 treatment group and showed a steady increase for approximately 10 d (Figure 3B).

### 3.3. Effects of L. plantarum HY7715 on Microbial Communities

**Figure 4:**
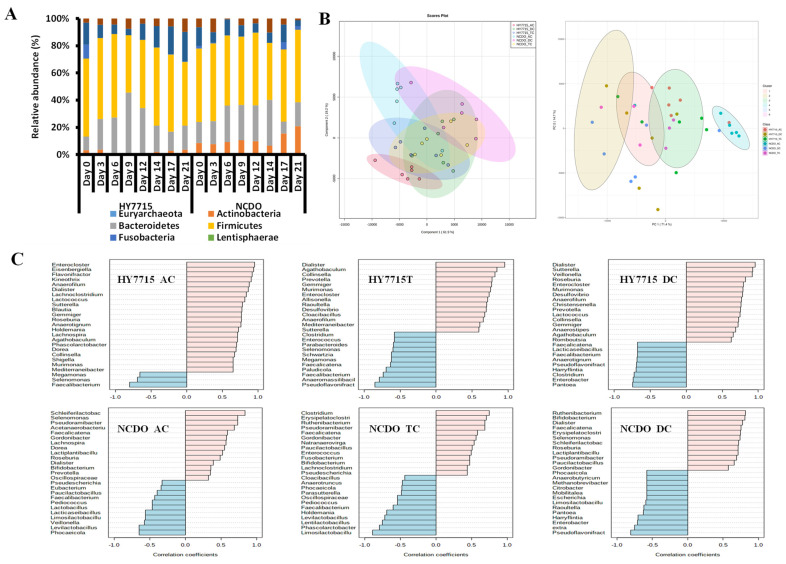
Microbiome change graph of simulated gut system according to HY7715 and NCDO1752 treatment. (**A**): Changes in the phylum level bacterial ratio (average value considering the volume of each site; AC = 300 mL, TC = 400 mL, DC = 325 mL). (**B**): Partial least squares-discriminant analysis (PLSDA, left) and K-means clustering (right) results by treatment group and site. (**C**): Correlation coefficient and list of strains showing an increase and decrease pattern according to the date of the HY7715 and NCDO treatment groups.

Changes in the microbiome following treatment with HY7715 and NCDO1752 at the phylum levels are shown in Figure 4A. The ratio of Bacteroidetes changed in the HY7715 treatment group and the ratio of Actinobaceria in the NCDO1752 treatment group changed. As a result of comparing the changes in microbiota according to the SGS part and the treatment date through the multivariate analysis, there was no principal component that could perfectly distinguish the difference between the groups (Figure 4B).

During the HY7715 treatment period, *Enterocloster*, *Eisenbergiella*, and *Flavonifractor* were the most abundant bacterial species in the AC portion, followed by *Dialister*, *Agathobaculum*, *Collinsella*, and *Prevotella* in the TC portion, and *Holdemania*, *Dialister*, and *Blautia* in the DC portion. Moreover, we observed concentrations of *Faecalibacterium* in the AC, *Pseudoflavonifract* in the TC, and *Klebdiella* in the DC. During the NCDO1752 treatment period, the bacterial species that mainly increased in the AC portion were *Schleiferilactobacillus*, *Selenomonas*, *Pseudoramibacter*, *Ruthenibacterium*, and *Bifidobacterium* (supporting the highest acetate content of the TC portion in the NCDO treatment group), and *Dialister* in the TC portion. Moreover, the bacterial species that increased in the DC portion were *Clostridium* and *Erysipelatoclostridium*. After the NCDO treatment, *Faecalibacterium* in the AC, *Pseudoflavonifractor* in the TC, and *Limosilactobacillus* in the DC showed decreasing tendencies. Although there was a slight difference in the increasing strains, the decreasing strains were similar (Figure 4C).

**Figure 5:**
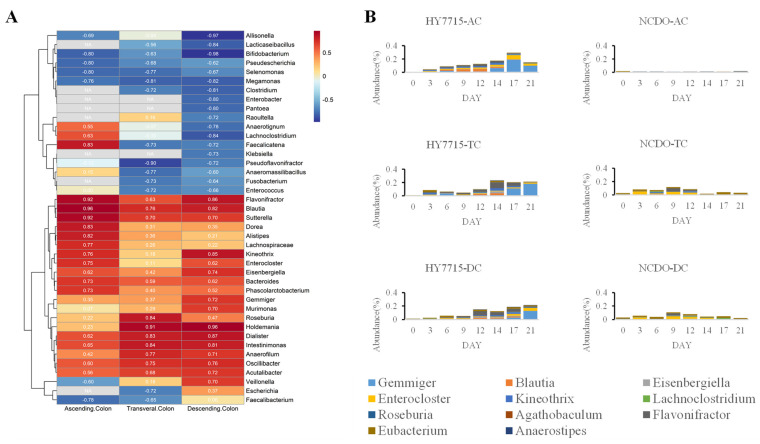
Microbial change associated with riboflavin and butyrate. (**A**): Heat map of the correlation coefficient of the strain, which showed a high correlation with increases in the riboflavin concentration (analysis proceeded with the results of the HY7715 treatment group, which showed a significantly increased riboflavin content). (**B**): Comparison of the sum of strains associated with butyrate production showing a high positive correlation in Figure 5A (HY7715 vs. NCDO1752).

The difference by region of the species known to be related to butyrate production was analyzed and it was confirmed that the ratio of strains such as Firmicutes, *Gemmiger*, *Blautia*, *Flavonifractor*, *Eisenbergiella*, *Kineothrix*, *Roseburia*, *Agathobaculum*, and *Eubacterium rectale* was relatively high in the HY7715 treatment group compared to NCDO1752. Figure 5A shows the heatmap analysis result of the microflora, which displayed a positive correlation with increasing riboflavin, as indicated by the correlation coefficient between these species and riboflavin. Bacteria related to butyrate production such as *Blautia*, *Flavonifractor*, and *Intestinimonas* showed a positive correlation (r > 0.75) with the riboflavin content. As a result of comparing the total strains known to produce butyrate for each part, it was confirmed that the relative ratio was high in all parts of the HY7715 treatment group, and the difference was the largest in the AC portion, which was directly injected (Figure 5B).

## 4. Discussion

In this study, we analyzed intestinal environment changes following the probiotic treatment of a human microbiome with HY7715 and NCDO1752 in a simulated gut system. Our primary observation after treatment was the difference in short-chain fatty acids between HY7715 and NCDO1752, which was attributed to butyrate and propionate variations. Additionally, by observing changes in the microbiome, we derived a correlation between SCFA production, the microbial community change, and characteristics of the treated strain in each group. In the NCDO1752 treatment group, the propionate concentration increased significantly (*p* < 0.05) at the AC and TC portions from the start of the treatment, whereas the acetate and butyrate concentrations tended to decrease over time. Acids such as lactate and acetate, produced by *Lactobacillus* controlled the pH of the intestine to inhibit the growth of harmful bacteria and help the growth of beneficial bacteria such as short-chain fatty acid-producing bacteria. It also selectively aided in the colonization of *Bifidobacterium* by producing prebiotics such as galactooligosaccharides that were used as food for Bifidobacteria. Bifidobacteria mainly produce acetate and propionate during metabolic pathways [32], and it was confirmed that the propionate concentration increased with the increase in Bifidobacteria in the NCDO1752 treatment group. In comparison with the NCDO1752 group, fluctuations in acetate and propionate did not show a significant increase in the HY7715 treatment group; however, the butyrate concentration showed a clear increase (*p* < 0.05). In the human intestine, butyrate is mainly produced in the phylum Firmicutes, especially *Faecalibacterium prausnitzii* and *Clostridium leptum* of the family Ruminococcaceae, and *Eubacterium rectale* and *Roseburia* spp. of the family Lachnospiraceae, and genera such as *Blautia*, *Flavonifractor*, *Roseburia*, and *Gemmiger*. The regional difference of the species known to be related to butyrate production was analyzed, and it was confirmed that the ratio of these butyrate production strains was relatively higher in the HY7715 treatment group than the NCDO1752 treatment group. The differences in the microbiomes after treating the samples with the same *Lactiplantibacillus species* (HY7715 and NCDO1752) were attributed to differences in the strain characteristics, such as fatty acid production and B2 production ability.

Short-chain fatty acids (SCFAs), including acetate, propionate, and butyrate, are the major metabolites produced by intestinal microflora during carbohydrate intake. SCFAs are produced primarily by intestinal microorganisms because the human genome does not encode a sufficient variety of lyases to effectively break down plant carbohydrates [33]. Moreover, SCFAs not only serve as an energy source for the host, but also have a significant impact on immunity. Butyrate is a short-chain fatty acid with anti-inflammatory and anticancer properties, and recent studies have reported an association with sarcopenia [34]. Interestingly, based on the recently published results for ameliorates sarcopenia of HY7715 [35], considering the changes in the butyrate concentration and butyrate-producing bacteria in the HY7715-treated group, the HY7715 intake has the potential to improve sarcopenia, but studies on the interaction between the microbiome and its metabolites should be conducted. Future studies using the simulated gut system require metabolomics-related research to understand comprehensive changes in complex metabolites produced by diverse microbial communities and influenced by their interaction.

Previous studies have reported the excellent gut survival rate and intestinal fixation of strain HY7715, which was used in this study to produce riboflavin. The results of our analyses confirmed that the production of riboflavin was maintained even when the treatment was stopped. Riboflavin is a water-soluble vitamin that is mainly consumed through dairy products, meat, and fish, and its deficiency is associated with anemia and cardiovascular disease. The risk of deficiency is high, particularly for people with an insufficient dairy and meat intake. In addition, Subramanian et al. (2019) reported that sodium butyrate induces the gene expression of the riboflavin transporter in the intestinal environment [36]. Therefore, the increase in butyrate and the confirmation of riboflavin production in the simulated colonic environment following the ingestion of HY7715 could further aid the absorption of riboflavin through probiotic intake. In particular, powdered probiotics can be administered to the elderly or vegetarians who have difficulty consuming dairy product or meat. It is predicted that this will help improve the intestinal environment by facilitating riboflavin absorption. Riboflavin is essential for energy-related metabolic pathways, and based on the evidence of low butyrate concentration in sarcopenia patients. The intake of probiotics with the overproduction of B2 such as HY7715 can provide beneficial effects to the human body by continuously producing B2 as well as the basic functionality of probiotics provided after the probiotics colonize in the intestine. By conducting additional clinical trials, it is possible to study the effect of enhancing muscle strength to confirm butyric acid production in the body for reducing muscle loss. Accumulating evidence has revealed a link between the intestinal microbiota composition and human health. Intestinal permeability is constantly changing depending on the diet and intestinal environment, and the firmness of the intestinal barrier is essential to prevent the excessive translocation of inflammatory molecules such as LPS [37]. Gut microorganisms affect the integrity of intestinal epithelial cells, leakage of inflammatory factors, and barrier dysfunction [38]. Butyrate has been reported to enhance the intestinal barrier by facilitating a tight junction assembly [39]. Based on the results of the increased butyrate content by the HY7715 treatment, we conducted a cell experiment using an SGS culture supernatant. However, significant results about enhancing the tight junction were not obtained. The use of a continuous simulated gut system in this study allowed for the accurate control of the intestinal environment, such as the pH and nutritional restriction. It is impossible to perfectly mimic the absorption processes of metabolites or fatty acids that occur in the human large intestine, but we expect that the study of the intestinal microbiome will be more realistic if organoids are grafted. To secure the reliability of probiotic research using SGS, accumulated research results are required compared with studies on animal and human application studies. Using Lab-on-a-chip technology, it is possible to study gene expression changes in human cells through real-time monitoring using gut, liver, and brain chips [40,41,42]. Incorporating these research tools, the simulated gut system is expected to have the potential to be used as a research tool close to animal or human experiments. In addition, using this technology, research on simulated gut systems can be conducted in actual clinical tests that are typically avoided due to human toxicity or disease concerns [43].

## 5. Conclusions

Through comparative experiments using a *L. plantarum*-type strain, we confirmed the effects of the *L. plantarum* HY7715 strain on the intestinal microbiome, which was previously reported to have a high gut survival and intestinal fixation rate as well as riboflavin overproduction. A large intestine-mimicking system (which can mimic the chemical and microbiological environment of the small intestine) used for probiotic and prebiotic screening was used in this study. Based on our analyses, we confirmed that HY7715 could alter intestinal microflora through the production of butyrate and riboflavin; thus, improving the intestinal environment. Based on our results, we expect that the colon-mimicking system will serve as an effective screening method for studying the interaction between the microbiome and probiotics and will facilitate animal and in vivo experiments.

## Data Availability

Not applicable.
